# Polycaprolactone/Chitosan Composite Nanofiber Membrane as a Preferred Scaffold for the Culture of Mesothelial Cells and the Repair of Damaged Mesothelium

**DOI:** 10.3390/ijms23179517

**Published:** 2022-08-23

**Authors:** Hao-Hsi Kao, Chang-Yi Kuo, Darshan Tagadur Govindaraju, Kuo-Su Chen, Jyh-Ping Chen

**Affiliations:** 1 Division of Nephrology, Chang Gung Memorial Hospital at Keelung, Keelung 20401, Taiwan; 2 Department of Chemical and Materials Engineering, Chang Gung University, Kwei-San, Taoyuan 33302, Taiwan; 3 School of Medicine, College of Medicine, Chang Gung University, Kwei-San, Taoyuan 33303, Taiwan; 4 Department of Neurosurgery, Chang Gung Memorial Hospital at Linkou, Kwei-San, Taoyuan 33305, Taiwan; 5 Craniofacial Research Center, Chang Gung Memorial Hospital at Linkou, Kwei-San, Taoyuan 33305, Taiwan; 6 Research Center for Food and Cosmetic Safety, College of Human Ecology, Chang Gung University of Science and Technology, Taoyuan 33305, Taiwan; 7 Department of Materials Engineering, Ming Chi University of Technology, Tai-Shan, New Taipei City 24301, Taiwan

**Keywords:** mesothelial cells, chitosan, nanofibers, electrospinning, polycaprolactone

## Abstract

Mesothelial cells are specific epithelial cells lining the serosal cavity and internal organs. Nonetheless, few studies have explored the possibility to culture mesothelial cells in a nanostructure scaffold for tissue engineering applications. Therefore, this study aims to fabricate nanofibers from a polycaprolactone (PCL) and PCL/chitosan (CS) blend by electrospinning, and to elucidate the effect of CS on the cellular response of mesothelial cells. The results demonstrate that a PCL and PCL/CS nanofiber membrane scaffold could be prepared with a comparable fiber diameter (~300 nm) and porosity for cell culture. Blending CS with PCL influenced the mechanical properties of the scaffold due to interference of PCL crystallinity in the nanofibers. However, CS substantially improves scaffold hydrophilicity and results in a ~6-times-higher cell attachment rate in PCL/CS. The mesothelial cells maintain high viability in both nanofiber membranes, but PCL/CS provides better maintenance of cobblestone-like mesothelial morphology. From gene expression analysis and immunofluorescence staining, the incorporation of CS also results in the upregulated expression of mesothelial marker genes and the enhanced production of key mesothelial maker proteins, endorsing PCL/CS to better maintain the mesothelial phenotype. The PCL/CS scaffold was therefore chosen for the in vivo studies, which involved transplanting a cell/scaffold construct containing allograft mesothelial cells for mesothelium reconstruction in rats. In the absence of mesothelial cells, the mesothelium wound covered with PCL/CS showed an inflammatory response. In contrast, a mesothelium layer similar to native mesothelium tissue could be obtained by implanting the cell/scaffold construct, based on hematoxylin and eosin (H&E) and immunohistochemical staining.

## 1. Introduction

The mesothelial cells play a key role in the mesothelium, by forming a smooth surface over internal organs, among their diverse biological functions and physiological roles. Although they are derived from the mesoderm, mesothelial cells demonstrate phenotypes from both epithelial and mesenchymal cells, and produce many growth factors, cytokines and extracellular matrix (ECM) components, as well as showing anti-inflammatory and immunomodulatory properties [1]. In the mesothelium layer, the cells rest on a thin basement membrane with predominantly flattened, squamous-like morphology [2]. The mesothelium monolayer from mesothelial cells forms a lining of the serosal cavities (pleural, pericardial and peritoneal) and the organs contained within these cavities. The major function of the mesothelium is as a protective non-adhesive surface; nonetheless, it is also involved in transport, inflammation, tissue repair, coagulation, and fibrinolysis etc. During peritoneal dialysis, dialysis liquids with a high glucose level, high molality and low pH may induce structural and functional peritoneal damage, and long-term exposure to these liquids may result in mesothelial cell atrophy and peritoneal sclerosis [3]. Although mesothelial regeneration could occur with cell migration from the wound edge or the attachment and incorporation of free-floating mesothelial cells from the serosal fluid onto the wound surface, impaired healing and cell transformation usually result in serosal adhesion formation. Mesothelial cell transplantation could be used in chronic renal failure patients receiving peritoneal dialysis to combat the decreased peritoneal dialysis efficacy [4]. Historically, mesothelial cell transplantation has been attempted by seeding cells in synthetic grafts or biological scaffolds, by the injection of suspended cells into the serosal cavity, or by cell sheet technology [5]. For autologous mesothelial cell transplantation, the intraperitoneal injection of suspended mesothelial cells into the serosal cavity has been used in patients with chronic renal failure under peritoneal dialysis therapy, by implanting autologous peritoneal mesothelial cells to coat the damaged tissue after cell culture and congelation [6]. Similarly, the intraperitoneal transplantation of a mesothelial cell suspension was proven to be effective to prevent postoperative abdominal adhesion formation in an animal model [7]. Comparing epithelial-like or fibroblastic-like mesothelial cells cultured from peritoneal dialysis patients in a peritoneal injury mice model, the transplantation of epithelial-like cells can prevent peritoneal adhesion, but not in the case of transplanting fibroblastic-like cells [8]. This study underscores the importance of the function and the phenotype of mesothelial cells. Undoubtedly, it is imperative to adopt a suitable cell culture environment in vitro, which can preserve the mesothelial phenotype for the effective repair of damaged mesothelium using mesothelial cell transplantation.

Tissue engineering as a method for the fabrication of functional tissues uses a three-dimensional (3D) scaffold as a structural base for cell attachment and proliferation [9]. The development of scaffolds from biomaterials showing degradability, biocompatibility and good mechanical properties has progressed considerably recently. A feasible approach for mesothelial cell transplantation can be envisioned by seeding cells in a scaffold. With the aim to develop a transplantable artificial peritoneum, an artificial peritoneum was constructed by entrapping fibroblasts and mesothelial cells in a collagen gel, which was later transplanted to peritoneal defects in rats [10]. Histological examination revealed that an artificial peritoneum, which consisted of a flat mesothelial monolayer upon a stromal matrix, adapted well to the host by preventing adhesion. Using this approach, mesothelial cells have been used in postoperative adhesion models by seeding mesothelial cells on a mixture of type I collagen and fibroblasts [11]. Similarly, in order to prevent adhesion formation in a rat intestinal hernia model, mesothelial cell sheets were cultured on fibrin gels [12]. Constructs seeded with mesothelial cells were grafted to an intestinal hernia model, and adhesion formation was not observed in the grafted rats. The grafted mesothelial cells remained at the host tissue site even after the degradation of the fibrin scaffold, indicating that the cultured mesothelial cell sheets can reduce postoperative complications caused by adhesion formation. In these studies, cell-seeding by loading a concentrated cell suspension onto the scaffold was found to be a reliable approach for establishing a mesothelial cell layer and maintaining the epithelial cell-like characteristics [13]. One of the major differences between using a scaffold for cell transplantation and injecting suspended cells is that the cell-seeded scaffold can be transplanted to the specific site of a lesion in order to create a mesothelial cell monolayer on the lesion in vivo. However, a scaffold-free approach was reported using the cell sheet technology, where a mesothelial cell sheet constructed from rodent peritoneal cells was obtained by two-step cell seeding, with a lower layer of peritoneal fibroblast cells and a monolayer of mesothelial cells as the upper layer [14]. These cell sheets were shown to successfully prevent postoperative peritoneal adhesions in a rat model.

Among the various methods for preparing 3D scaffolds, there has been considerable interest in using electrospinning to fabricate the scaffolds [15]. Electrospinning is a simple and convenient method for producing micro/microfibers with fiber diameters ranging from tens of nanometers to micrometers [16]. Electrospinning allows the production of porous nanofibrous membranes with specific features, including 3D morphology, large surface area-to-volume ratio, and high porosity. The structure of an electrospun nanofiber membrane mimics the nanofibrous environment provided by the extracellular matrix (ECM) components, which provides a natural base for cell adhesion, migration and proliferation [17]. Hence, we aim to fabricate macroporous nanofiber membranes as a scaffold for the culture of mesothelial cells. Considering scaffold base materials, both synthetic and natural polymers can be fabricated into nanofiber membranes by electrospinning. Polycaprolactone (PCL) is a synthetic polymer of interest in many electrospinning studies, which is a highly hydrophobic semi-crystalline polyester with good mechanical properties and long biodegradation times [18]. Chitosan (CS) is a natural carbohydrate polymer which has also been used for the production of fibrous membranes by electrospinning [19,20]. Specifically, CS alone or its derivatives have been widely used in biomedical applications due to their biocompatibility and biodegradability [21,22]. There have been several studies evaluating the cytocompatibility of CS, from which CS was found to be nontoxic and to support the growth of different cell lines [23,24]. A polymer blend may combine the advantages offered by its components. Therefore, a nanofiber membrane scaffold composed of composite nanofibers by the electrospinning of a polymer blend is a preferred method by which to fabricate a tissue engineering scaffold. It is expected that an electrospun nanofiber membrane containing both CS and PCL can exhibit the physicochemical properties from both polymers, such as providing biochemical cues from the natural polymer CS, as well as offering mechanical strength from the synthetic polymer PCL.

In this study, we fabricate electrospun nanofibers from PCL and blends of PCL/CS. We compare the cellular response of mesothelial cells in the nanofiber membrane scaffold in vitro, and use the preferred PCL/CS nanofiber membrane scaffold to repair damaged mesothelium in rats.

## 2. Results

### 2.1. Characterization of PCL and PCL/CS Electrospun Nanofiber Membranes

**Figure 1:**
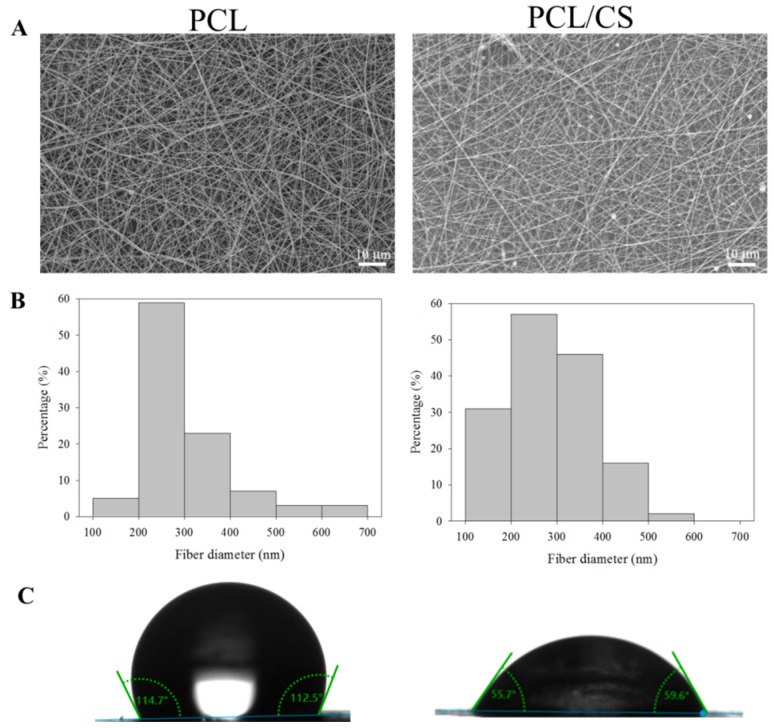
The scanning electron microcopy (SEM) micrograph ((**A**), bar = 10 μm), fiber diameter distribution (**B**), and water contact angle (**C**) of PCL and PCL/CS nanofiber membranes.

The PCL and PCL/CS nanofiber membranes were prepared successfully with 224 ± 5 and 218 ± 5 µm thicknesses, respectively. The morphology of the nanofibers was observed under scanning electron microscopy (SEM) observation. As shown in Figure 1A, nanofibers with smooth surfaces without bead formation were found in the membrane scaffold with the pre-determined electrospinning conditions. The distribution of the fiber diameter is close to a normal distribution (Figure 1B), and the mean value of the fiber diameter is below 300 nm for both membranes, with no significance difference found between them (Table 1). The membrane revealed a macroporous structure from SEM observation, indicating that nutrients and metabolic wastes may pass through the pores, although the limited pore size may hinder cellular ingrowth. The water contact angle was measured in order to elucidate the hydrophilicity of the membrane scaffold (Figure 1C). After blending the PCL with CS, the mean value of the water contact angle substantially decreased from 113.6° to 60.9°, indicating that the membrane scaffold becomes more hydrophilic with increased wettability (Table 1). If we compare the mean values of the porosity and density, the PCL nanofiber membrane has lower porosity (68.3% vs. 74.2%) but higher density (0.484 vs. 0.478) than PCL/CS, although no significant difference was found for density (Table 1).

**Figure 2:**
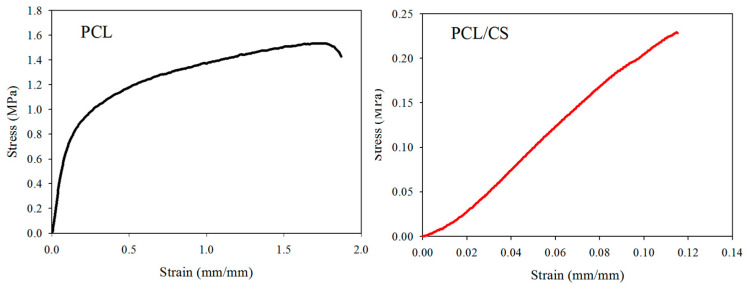
The typical tensile stress–strain curves of PCL and PCL/CS nanofiber membranes.

The mechanical properties of wet membrane scaffolds after immersion in phosphate buffered saline (PBS) for 24 h were studied, and typical stress–strain curves were produced by uniaxial tensile testing (Figure 2). As shown in Table 2, the ultimate tensile stress and ultimate tensile strain of the scaffold were reduced by blending PCL with CS. Indeed, the ultimate stress (or strain) of PCL was seven (or 14) times that of PCL/CS. Similarly, the Young’s modulus of PCL also decreased 3.4-fold after blending with CS.

**Figure 3:**
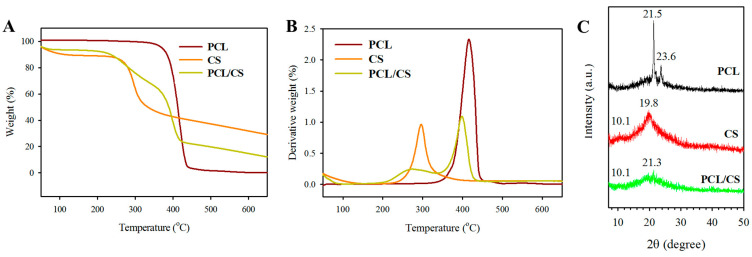
The thermogravimetric analysis (TGA) (**A**), derivative thermogravimetric analysis (DTA) (**B**) and X-ray diffraction (XRD) analysis (**C**) of PCL and PCL/CS nanofiber membranes. The CS polymer is used for comparison.

Thermogravimetric analysis (TGA, Figure 3A) and derivative thermogravimetric analysis (DTA, Figure 3B) were used to study the thermal properties of PCL and PCL/CS, as well as the effects of blending of PCL with CS. From the TGA curve, the PCL nanofiber membrane shows single-stage thermal degradation, starting at 342 °C and ending at 432 °C, with 0.1% residual weight at 650 °C due to its synthetic polymer nature. The DTA curve shows a single peak decomposition temperature at 416 °C. The CS polymer shows some weight loss before 100 °C, which may be assigned to water evaporation from the sample. A major weight loss is found between 216 and 370 °C for CS from TGA, with ~28% residual weight for this natural carbohydrate polymer. The peak decomposition temperature was 296 °C for CS from DTA. The PCL/CS nanofiber membrane depicts two-stage thermal degradation starting at 200 °C, and completes at 420 °C, with 13% residual weight primarily from CS. There are two decomposition peak temperatures: a broad peak starting as early as 275 °C due to CS, and a sharper second peak at 399 °C due to PCL. The residual weight of PCL/CS is close to the theoretical value (14%) calculated from the weight percentage of PCL (50%) and CS (50%) in the blend.

Figure 3C depicts the X-ray diffraction (XRD) analysis of a PCL nanofiber membrane, where a sharp crystalline peak at 21.5° (110) and a relatively low-intensity peak at 23.6° (200) were found due to the semi-crystalline nature of PCL polymer. The CS polymer shows broad diffraction peaks in the XRD pattern at 10.1° and 19.8°, indicating its amorphous nature. The PCL/CS nanofiber membrane shows amorphous peaks at 10.1° and a very broad peak at 21.3° from the merging of the characteristic peaks of PCL and CS, but with diminished peak intensity. Taken together, the XRD analysis indicates that the crystallinity of electrospun PCL nanofiber membrane was reduced by using a PCL/CS blend for electrospinning.

### 2.2. In Vitro Cell Culture

**Figure 4:**
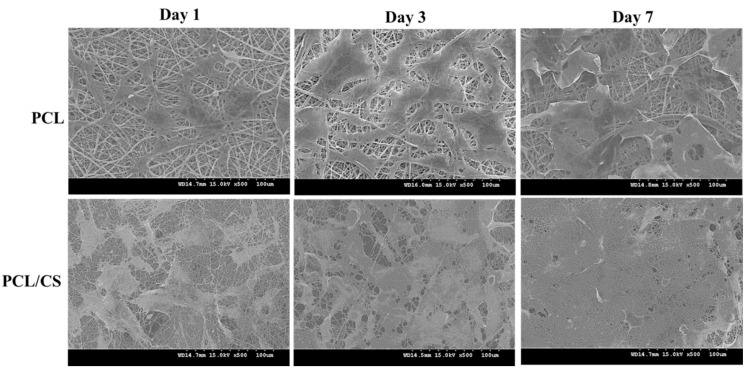
The scanning electron microcopy (SEM) images of cell-seeded PCL and PCL/CS nanofiber membranes after being cultured for 1, 3 and 7 days. Bar = 100 μm.

As seen in the SEM observations of cell-seeded membranes, the mesothelial cells were mostly polygonal in shape (Figure 4). By displaying a typical cobblestone phenotype, the attached mesothelial cells preserve their epithelial characteristics. Comparing the cell morphology on day 1 and 7, the mesothelial cells maintain their epithelial-like characteristics during cell growth, albeit that they become more elongated on day 7. Overall, the cell morphology observation from SEM supports the maintenance of the mesothelial phenotype in both nanofiber membrane scaffolds.

**Figure 5:**
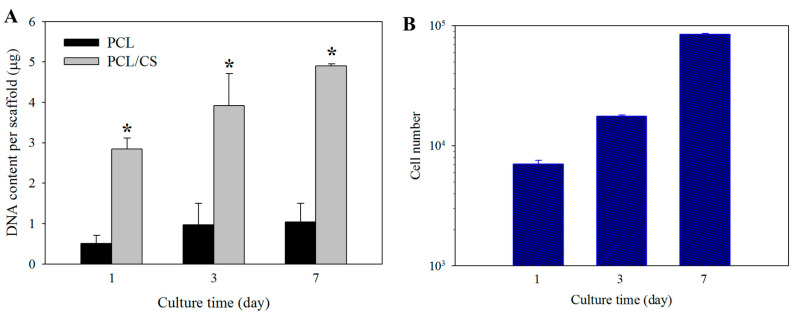
(**A**) Proliferation of mesothelial cells in PCL and PCL/CS nanofiber membranes from DNA assays. * *p* < 0.05 compared with PCL. (**B**) Proliferation of mesothelial cells on a petri dish from cell counting.

In order to determine cell proliferation, the cell number in PCL and PCL/CS was compared based on the DNA content per membrane scaffold (Figure 5A). The mesothelial cells steadily proliferated from day 1 to 7, with significantly higher DNA content in PCL/CS than in PCL at all time points. The difference in cell seeding efficiency between the membranes was determined from the DNA content on day 1, with PCL/CS showing 5.7 ± 0.5 folds of DNA contents compared with PCL. On days 3 and 7, the DNA contents of PCL/CS were 4.1 ± 1.3 and 4.7 ± 0.5 folds compared with PCL, respectively. For comparison, the cell proliferation rate when mesothelial cells are cultured on a petri dish is shown in Figure 5B.

**Figure 6:**
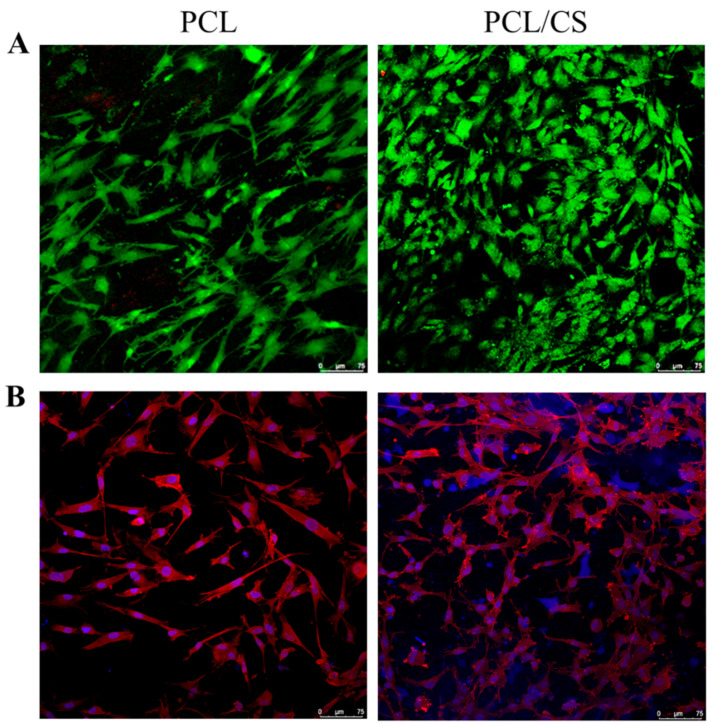
The confocal microscopy observation of mesothelial cells cultured in PCL and PCL/CS nanofiber membranes by live/dead ((**A**), bar = 75 μm) and nucleus/cytoskeleton staining ((**B**), bar = 75 μm). The live cells were stained green by calcein AM, and dead cells were stained red by ethidium homodimer-1 in (**A**). The actin cytoskeleton was stained red by phalloidin-rhodamine, and the cell nucleus was stained blue by Hoechst 33258 in (**B**).

Maintaining high viability is a key issue during the in vitro culture of mesothelial cells. Therefore, cell viability in the scaffold was evaluated by live/dead staining, and was examined by confocal microscopy. As shown in Figure 6A, stacked confocal images examined from the membrane surface show a higher number of viable cells emitting green fluorescence on day 7, with a negligible number of dead cells emitting red fluorescence. Judging from the distribution of green fluorescence, a general cobblestone cell morphology of the mesothelial cells is also supported. However, cells in PCL appear to exhibit a more elongated fibroblastic shape than in PCL/CS, implying that CS may contribute to the maintenance of the mesothelial phenotype better. Furthermore, more live cells were found within PCL/CS, to be consistent with the DNA analysis. The cytoskeleton expression of mesothelial cells in the scaffold was revealed from phalloidin-rhodamine staining, with cell nuclei counter-stained with Hoechst 33258 in blue, for confocal microscopy examination (Figure 6B). Although close-to-round-shaped nuclei were observed in both membranes, there appears to be a difference in cytoskeleton organization, as revealed from the red actin-rich microfilaments arranged in stress fibers. More cells with a cobblestone-like appearance were shown in the confocal image of PCL/CS, while the confocal image of PCL shows a greater non-epithelial fibroblast-like cell population.

**Figure 7:**
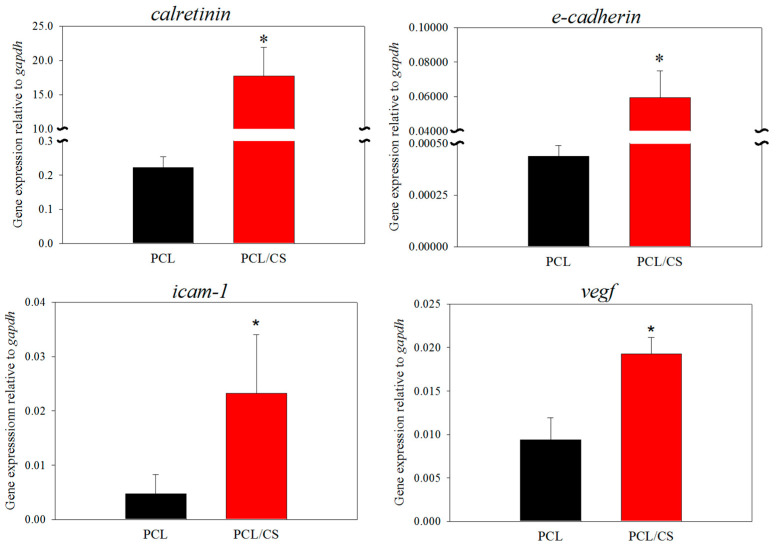
The relative gene expression level of calretinin, E-cadherin, intercellular adhesion molecule (ICAM-1) and vascular endothelial factor (VEGF) by mesothelial cells was determined by quantitative real-time polymerase chain reaction (qRT-PCR) after culture for 7 days in PCL and PCL/CS nanofiber membranes. Glyceraldehyde 3-phosphate dehydrogenase (GAPDH) was used for the quantification. * *p* < 0.05 compared with PCL.

In order to assess the influence of CS on the mesothelial characteristics of cultured mesothelial cells, we performed a gene expression analysis of the mesothelial marker genes. The relative gene expression of E-cadherin, calretinin, vascular endothelial factor (VEGF), and intercellular adhesion molecule (ICAM-1) were studied using a quantitative real-time polymerase chain reaction (qRT-PCR). The relative mRNA levels of all of the genes were quantified relative to glyceraldehyde 3-phosphate dehydrogenase (GAPDH). As shown in Figure 7, the gene expression level of all of the mesothelial marker genes (E-cadherin, calretinin, VEGF and ICAM-1) is upregulated in the presence of CS. The mesothelial cells cultured in PCL/CS nanofiber membrane show significantly higher relative mRNA level compared to PCL, regardless of the type of gene under study. Nonetheless, the extent of gene expression upregulation is much pronounced for *calretinin* and *e-cadherin*, where ~196 and ~80 fold increases of the gene expression level are found for cells cultured in PCL/CS vs. those cultured in PCL.

**Figure 8:**
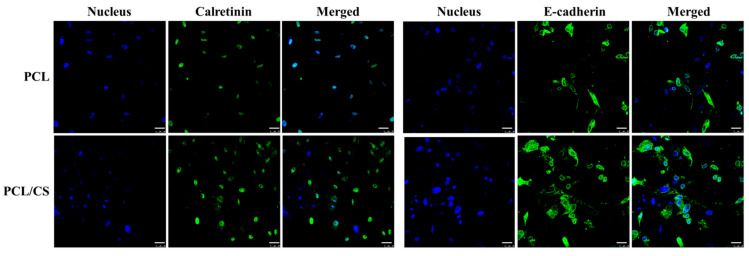
The immunofluorescence (IF) staining of calretinin and E-cadherin produced by mesothelial cells cultured in PCL and PCL/CS nanofiber membranes for 7 days. The proteins were stained green using a fluorescein (FITC)-conjugated secondary antibody that can bind with the primary antibody against calretinin or E-cadherin. The nuclei were stained blue by Hoechst 33258. Bar = 25 μm.

In order to confirm the elevated expression of mesothelial maker genes, the extent of marker protein synthesis by mesothelial cells was observed by immunofluorescence (IF) staining, with the cell nucleus being identified by nuclear staining (Figure 8). As is consistent with the trend of gene expression levels, the confocal image reveals more green signal associated with the FITC-labelled antibody when PCL/CS is used for the cell culture. As mesothelial cells in PCL/CS appear to have a higher synthesis rate of these mesothelial marker proteins, we carried out a semi-quantitative analysis of the green-fluorescence-stained areas in the IF images, for the comparison of their protein synthesis ability. The area percentage of the green fluorescence signal associated with calretinin is 5.73 ± 0.02% for PCL/CS and 3.45 ± 0.37% for PCL, while for E-cadherin it is 16.85 ± 1.02% for PLC/CS and 7.58 ± 0.26% for PCL. The significant enhancement of mesothelial marker protein production supports the use of PCL/CS to provide a better milieu for preserving the mesothelial phenotype.

### 2.3. In Vivo Study

**Figure 9:**
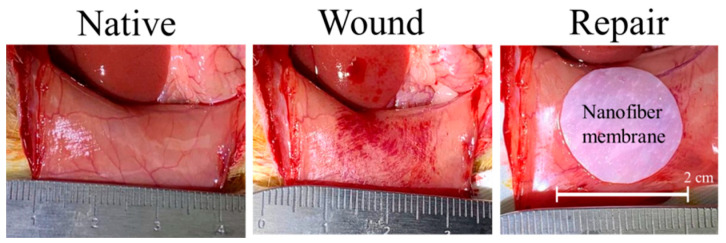
A wound was created on the native parietal peritoneum after abrading, followed by repair with a 2-cm PCL/CS nanofiber membrane, with or without mesothelial cells.

Due to the better mesothelial cellular response to PCL/CS nanofiber membrane in vitro, this scaffold was chosen for the in vivo study. As an animal model for peritoneal mesothelial repair, rat parietal peritoneum was abraded to create a 15-mm diameter wound, followed by covering with a 2-cm diameter PCL/CS nanofiber membrane, without suturing (Figure 9). Both cell-free PCL/CS membrane (acellular group) and PCL/CS membrane seeded with mesothelial cells and followed with 7-day in vitro cell culture (cellular group) were used for the study. During the 7-day observation period post-implantation, we did not observe any animal disability, infection or death. The nanofiber membrane was well recognized on the abdominal wall, and did not adhere to any internal organs. The formation of new tissues was scarce, and there was no ecchymosis around the scaffold, albeit that local edema was noted. 

**Figure 10:**
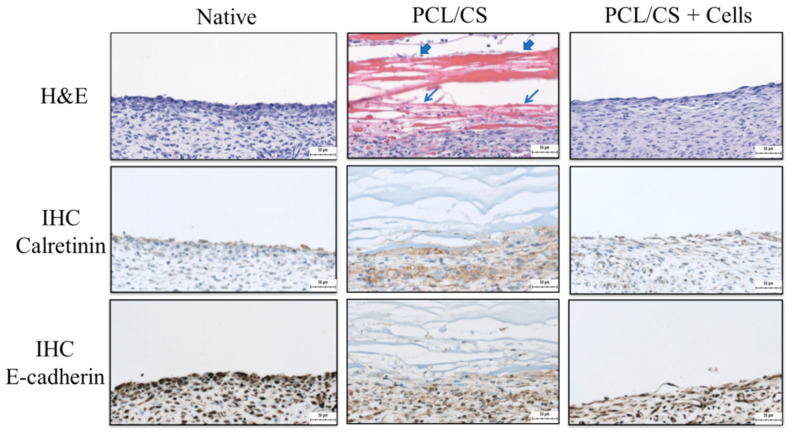
The hematoxylin and eosin (H&E) and immunohistochemical (IHC) staining of calretinin and E-cadherin of the mesothelium tissue 7 days post-implantation (bar = 50 μm). The abraded parietal peritoneum was repaired with PCL/CS or PCL/CS + cells as the acellular and the cellular groups, respectively. The PCL/CS + cells sample is prepared by culturing mesothelial cells in PCL/CS membrane scaffold for 7 days in vitro before implantation. The native mesothelium is used for comparison on the left. The arrowheads indicate residual membrane filaments, and arrows indicate the inflammatory cell layer in PCL/CS.

The repaired mesothelium was retrieved and examined histologically 7 days post-implantation. From histological examination, the hematoxylin and eosin (H & E) staining showed typical elongated, flattened mesothelial cells on the surface of recovered mesothelium in the PCL/CS + cells (cellular) group (Figure 10). From the immunohistochemical (IHC) staining of E-cadherin and calretinin, key mesothelial marker proteins produced by transplanted mesothelial cells were clearly identified by the stained brown color around cell nuclei in the cellular group. This endorses the continuous production of mesothelial marker proteins by mesothelial cells in the transplanted allograft, which can contribute to the repair of damaged mesothelium. In the PCL/CS (acellular) group, some residual membrane filaments showing a red color could be identified, which are adhered to the wound from the H&E staining image (Figure 10). A thick cell layer devoid of mesothelial cell morphology was noted underneath the residual membrane layer from H&E staining, which did not show the synthesis of the mesothelial marker protein calretinin and E-cadherin from IHC staining. This may arise from the inflammatory response when the PCL/CS scaffold alone was used for mesothelium repair. Overall, a mesothelium layer similar to the native mesothelium tissue was noted from the PCL/CS + cells (cellular) group, from both H&E and IHC staining. However, the loose structure shown from the outer cell layer in the PCL/CS + cells (cellular) group may arise from insufficient implantation time.

## 3. Discussion

Mesothelial cells play a critical role in the mediation of inflammation through their ability to synthesize growth factors, ECM proteins and hyaluronan [25,26]. They are fundamental for the maintenance of serosal integrity and homeostasis, and play an important role in normal serosal repair after injury [27]. Peritoneal mesothelial cells are epithelial-like cells resting on a thin basement membrane lining the entire abdominal cavity, from which a cell monolayer (mesothelium) is formed as a non-adhesive protective barrier [28]. The boundaries between mesothelial cells consist of delicate junctional complexes that are crucial for the maintenance of a tight semi-permeable diffusion barrier [29]. Being part of the peritoneum, mesothelial cells participate in its structural and functional activity, and play an important role during the repair of damaged mesothelium [30].

In this study, we use electrospun nanofiber membrane scaffold for the seeding of mesothelial cells, and intend to repair damaged mesothelium with this cell/scaffold construct in vivo. The peritoneal membrane is composed of a single layer of flattened mesothelial cells placed on the surface of a thin layer of collagenous tissue [31]. As the basal lamina provides a porous ECM structure, the structure of a nanofiber membrane scaffold can mimic the topology and porosity in the basal lamina. For mesothelium tissue regeneration, the limited pore size of the electrospun membrane may prevent efficient cell ingrowth for attached mesothelial cells. However, as a mesothelial layer contains only a few layers of mesothelial cells, the attachment and proliferation of a single layer of mesothelial cells in the nanofiber membrane scaffold may suffice, pertaining to high cell viability and the maintenance of the mesothelial phenotype. The scaffold porosity is one of the key metrics of a tissue engineering scaffold, and is inversely related to the scaffold density. The macroporous nature of the PCL and PCL/CS nanofiber membrane scaffold is expected to facilitate cell proliferation (Table 1), which can also provide adequate gas and nutrient exchange during peritoneal wound healing.

PCL has been widely used in tissue engineering; it is a hydrophobic semi-crystalline synthetic polyester polymer. Therefore, a nanofiber membrane fabricated from PCL represents a mechanically robust scaffold to repair the peritoneum tissue. After blending PCL with CS, the inferior mechanical properties of PCL/CS can be explained using XRD analysis. As evidenced by the decreased XRD peak intensities, the blending of PCL with CS appears to interfere with its degree of crystallinity in the nanofibers, and appears to influence the crystalline nature of the scaffold. This is consistent with a previous report that changes in crystallinity may affect the mechanical properties of nanofiber membranes during tensile mechanical testing [32]. However, the decrease of the crystallinity after polymer blending suggests the homogeneous mixing of both polymers in the blend at a molecular level [33]. Nevertheless, the mechanical properties of PCL/CS (Table 2) can still meet the need for mesothelium repair, with the ultimate strain of peritoneum tissues ranging from 26% to 31%, the ultimate stress ranging from 0.37 MPa to 0.58 MPa, and the Young’s modulus ranging from 0.09 to 1.01 MPa [34].

Even though PCL has good mechanical properties and biodegradability, it is endowed with high hydrophobicity and a lack of favorable cellular response [35]. One way to overcome such limitations is to blend PCL with a hydrophilic polymer, for example CS, as was used in this study. CS is a natural, nontoxic, biocompatible, and biodegradable polymer with anti-microbial activity [36,37]. Recent studies further indicate that CS or its derivatives are novel scaffold materials for tissue engineering which can provide a positive cellular response [38,39,40]. In order to combine the advantages offered by PCL with those from CS, a PCL/CS composite nanofiber membrane was fabricated in this study, and was shown to be beneficial for the attachment, marker gene expression and protein synthesis of mesothelial cells. From contact angle measurement, which is an index of substrate wettability affecting cell attachment, the PCL/CS nanofiber membrane shows a contact angle about half that of PCL (Table 1). With this change, a greater number of mesothelial cells were found to be adhered to PCL/CS, as expected. From a DNA content analysis, a 5.7-fold increase of the attached cell number was found for PCL/CS over PCL on day 1. Furthermore, continuous cell proliferation was found for both scaffolds with a time-lapsed increase of DNA content, suggesting that the nanofiber membrane scaffold is beneficial for the growth of mesothelial cells in vitro. Concerning the effect of CS on the cell proliferation rate, the blending of CS with PCL may influence cell growth, where the increase of DNA content from day 1 to day 3 is 1.90 fold for PCL, in contrast to 1.38 fold for PCL/CS. It is worth noting that as one of the surface properties, the wettability of a scaffold may also influence the cell growth rate within the scaffold [41].

The culture of mesothelial cells in PCL/CS leads to the significant upregulation of mesothelial marker genes, including calretinin, E-cadherin, ICAM-1 and VEGF, from gene expression analysis using qRT-PCR. Calretinin is a calcium-binding protein involved in calcium signaling, which is synthesized in mesothelial cells in addition to certain neural tissues [42]. The exact function of calretinin is not well-defined, but it may play a role in the cell cycle [43]. Nonetheless, several immunohistochemical studies have suggested that calretinin is a very useful marker for cells of mesothelial lineage [42]. Mesothelial cells express several cell-adhesion molecules, including E-cadherin and ICAM-1 [44]. E-Cadherin is a calcium-dependent transmembrane epithelial protein promoting intercellular adhesion [45]. It appears to have a central role in the control of epithelial-to-mesenchymal transition, as the loss of E-cadherin expression is correlated with the ability of epithelial cells to adopt a mesenchymal migratory and invasive phenotype [46]. The expression of the adhesion molecule ICAM-1 is constitutive in mesothelial cells [47]. It appears to be a potential marker that can discriminate between mesothelial cells and fibroblasts [48]. The importance of ICAM-1 in leucocyte transmigration across the mesothelium has been demonstrated in pleural and peritoneal cells [49]. It was reported that neutrophil migration requires ICAM-1 expression in addition to the activation and release of cytokines from the mesothelial cells [50]. The incubation of cells with soluble ICAM-1 significantly reduces neutrophil transmigration [51]. The vascular endothelial growth factor (VEGF) is a potent proangiogenic factor involved in endothelial cell proliferation and vascular permeability [52]. VEGF is considered to be a main growth factor for mesothelial cells, with its increased production stimulating the growth of mesothelial cells [53]. Furthermore, the local production of VEGF by transitional mesothelial cells appears to play an important role during the process of peritoneal angiogenesis [54]. Taken together, the gene expression analysis indicates that CS can upregulate all mesothelial marker genes, implicating the better maintenance of the mesothelial cell phenotype in PCL/CS.

In order to confirm the gene expression patterns, the production of calretinin and E-cadherin was evaluated from IF staining. The production of these mesothelial marker proteins from mesothelial cells in both nanofiber membranes is evident, but with distinctive features. The mesothelial-specific proteins were found to be distributed within the electrospun membrane, associating with mesothelial cells which were identifiable from nuclear staining. Nonetheless, as is consistent with the gene expression, the production rates of both calretinin and E-cadherin were found to be elevated in PCL/CS.

The mesothelial cells form a monolayer, known as the mesothelium, which is a low-friction, non-adhesive surface and selective barrier, and regulates the transport of fluid and solutes between the circulation, the interstitial space and the body cavities [55]. From H&E staining, many inflammatory cells were found to infiltrate the upper layer of damaged mesothelium when the wound is covered with a PCL/CS nanofiber membrane in the acellular group. In contrast, for the cellular group, when the wound is covered with PCL/CS + cells, the uppermost layer of the damaged mesothelium shows a mesothelial cell layer closely resembling that of a native mesothelium. The IHC staining also endorsed the formation of a mesothelium layer similar to that of the native mesothelium in the cellular group, where transplanted mesothelial cells can secrete abundant key mesothelial marker protein calretinin and E-cadherin. The uppermost layer of the parietal peritoneum surface is where mesothelial cells should be distributed, as they will not perform their normal physiological functions elsewhere. After implantation, a normal physiological manifestation of the mesothelium was noted, similarly to mesothelial cells at the visceral and parietal contact with close-to-monolayer distribution. It is expected that the repaired mesothelium will be re-modelled, and that a functional mesothelium will be developed from the allograft mesothelial cells, after scaffold-mediated cell transplantation to the wound for the repair of the damaged mesothelium.

## 4. Materials and Methods

### 4.1. Materials

Chitosan (CS, molecular weight = 60,000–120,000 Da, degree of deacetylation = 70%), polycaprolactone (PCL, molecular weight = 80,000 Da) and polyethylene oxide (PEO, molecular weight = 2,000,000 Da) were purchased from Sigma-Aldrich (St. Louis, MO, USA). The live/dead viability/cytotoxicity kit for mammalian cells, phalloidin-tetramethylrhodamine B isothiocyanate (phalloidin-TRITC) for F-actin staining, Hoechst 33258 for nuclear staining, fetal bovine serum (FBS), Dulbecco’s modified Eagle medium (DMEM), trypsin-EDTA and penicillin/streptomycin solution were obtained from Life Technologies (Carlsbad, CA, USA). The primers for PCR were supplied by Tri-I Biotech, Inc. (Taipei, Taiwan).

### 4.2. Preparation of the Nanofiber Membranes by Electrospinning

The spinning solution for the PCL nanofiber membrane was a 12% (*w*/*w*) PCL solution prepared in chloroform/methanol with a 7/3 volume ratio. The spinning solution for the PCL/CS nanofiber membrane was 2% (*w*/*w*), PCL/2% (*w*/*w*), CS/0.25% (*w*/*w*), PEO prepared in 90% (*v*/*v*) acetic acid. The electrospinning was carried out with a blunt needle (21 gauge) fitted to a 10-mL syringe. The syringe was mounted on a syringe pump (KD Scientific Co., Holliston, MA, USA) and the polymer solution was drawn horizontally from the needle tip to a grounded collector (covered with an aluminum foil), placed 10 cm from the needle tip. By providing an electrostatic force from a high-voltage supply at 15 kV (or 23 kV) between the tip and the grounded collector, the polymer solution was ejected from the syringe at 0.9 mL/h (or 0.6 mL/h) for PCL (or PCL/CS) nanofiber membranes.

### 4.3. Characterization of the Nanofiber Membranes

The morphology of the nanofibers in the electrospun membrane was examined with a scanning electron microscope (SEM, JEOL JSM-7500F, Tokyo, Japan). The fiber diameter was estimated from 5 SEM images using ImageJ software, with 20 fibers being randomly chosen from each image. The water contact angle of a nanofiber membrane was determined by the sessile drop method using an FTA-125 contact angle machine (First Ten Angstroms, Portsmouth, VA, USA). After 3 s of dripping 2 μL distilled water onto the membrane, the contact angles of the droplets were analyzed from both sides. The contact angles from three samples were reported. The porosity was measured by a liquid displacement method based on Archimedes’ principle using a specific-gravity bottle filled with ethanol, using the ability of ethanol to completely fill the pores of a nanofiber membrane. The density was calculated by determining the weight of a 2-cm-diameter disk-shaped membrane, and we divided the weight by the volume of the membrane. The volume was calculated from the thickness of the membrane, which was determined with a digital micrometer beforehand. The porosity and density were the mean value of three samples. A Tinius Olsen mechanical testing machine (H1KT, Horsham, PA, USA) was used to evaluate the mechanical properties of the membrane using a 10-N load cell. The uniaxial tensile properties of the membrane were obtained at a 5 mm/min elongation rate. A 1 cm × 5 cm test sample was mounted vertically between two mechanical grips, leaving a 3-cm gauge length for mechanical loading. The ultimate tensile stress, ultimate tensile strain, and Young’s modulus were obtained from the stress–strain curve. The thermal stability of the nanofiber membrane was characterized using thermogravimetric analysis (TGA) and derivative thermogravimetric analysis (DTA) with TGA Q50 from TA Instruments (New Castle, DE, USA). The temperature for the analysis was set from room temperature to 700 °C under a nitrogen atmosphere at a heating rate of 10 °C/min. For the X-ray diffraction (XRD) analysis, a D2 PHASER X-ray diffractometer from Bruker (Billerica, MA, USA) was used with a Cu-Kα X-ray source (λ = 1.5406 Å) from a 10 to 50 degree 2θ angle.

### 4.4. Isolation of the Mesothelial Cells

The mesothelial cells were harvested from the lower abdomen of Sprague-Dawley (SD) rats after sterilizing the abdomen wall. The skin was picked up with a forceps on the abdomen wall, and the skin was gently cut with a blade to separate it from the peritoneum. The peritoneum was diced into 2 × 2 cm pieces using scissors in a sterile procedure. The diced peritoneum sample was washed extensively with phosphate-buffered saline (PBS), and the extracellular matrix (ECM) was digested with 0.2% collagenase for 30 min in a shaking water bath operating at 20 rpm and 37 °C. After digestion, the peritoneum was discarded and the liquid was filtered through a 70-μm-pore-size filter to remove the debris. The enzyme activity was neutralized by adding 20 mL cell culture medium (90% DMEM high glucose and 10% FBS), and was centrifuged at 100× *g* for 5 min to obtain a high-density cell pellet. The supernatant was discarded, and the cell pellet was re-suspended in cell culture medium and incubated at room temperature.

### 4.5. In Vitro Cell Culture

A disk-shaped membrane (12-mm diameter) was sterilized with 75% ethanol for 24 h and rinsed twice with PBS before being placed in a 24-well cell culture plate for cell seeding. An aliquot of 10 μL cell suspension (1 × 10^7^ cells/mL) was seeded onto the surface of the membrane and incubated at 37 °C for 2 h to allow cell adhesion, followed by adding 1 mL culture medium. The cells were cultured at 37 °C in 5% CO_2_ with a medium change every three days.

### 4.6. Scanning Electron Microscopy (SEM) Analysis

For the SEM analysis, the cell-seeded membrane was removed from the cell culture plate and fixed with glutaraldehyde for 3 h at room temperature. All of the samples were washed 3 times with PBS and distilled deionized water, and were treated with increasing concentrations of ethanol from 50% to 99.5% for dehydration. The samples were air dried and sputter coated with gold for observation with a Hitachi S-3000N scanning electron microscope (SEM, Tokyo, Japan).

### 4.7. Cell Proliferation

The cell-seeded membranes were harvested at predetermined times and digested for 24 h in a lysis solution (55 mM sodium citrate, 150 mM sodium chloride, 5 mM cysteine, 5 mM EDTA, and 0.2 mg/mL papain) at 60 °C. The solution was centrifuged and the DNA content in the supernatant was quantified using Hoechst 33258 in an enzyme-linked immunosorbent assay (ELISA) reader (Excitation: 360 nm, Emission: 460 nm). A standard curve constructed from calf thymus DNA was used. For comparison, cell proliferation in a petri dish was carried out by seeding 5 × 10^3^ cells on a 35-mm petri dish. After different culture times, the cell number in each dish was determined directly by counting with a hemocytometer after detaching the cells with 0.05% trypsin-EDTA.

### 4.8. Quantitative Real-Time Polymerase Chain Reaction (qRT-PCR)

The expression of mesothelial marker genes was analyzed by quantitative real-time polymerase chain reaction (qRT-PCR). Total RNA from each sample was isolated using Trizol, and was dissolved in DEPC-treated water for the determination of the RNA concentration at 260 nm (OD_260_). The RNA quality was verified from OD_260_/OD_280_ measurements. The complementary DNA was synthesized in a Maxime RT premix kit (Intron Biotechnology, Seoul, Korea) according to the standard procedures. Glyceraldehyde-3-phosphate dehydrogenase (GAPDH) acted as a housekeeping control. Amplification was conducted for 45 cycles in a thermo cycler. Each cycle consisted of 10 min at 95 °C for denaturation, 30 s at 95 °C for annealing, and 1 min at 60.9 °C (calretinin) or 67.1 °C (E-cadherin, GAPDH, ICAM-1 and VEGF) for extension. Real-time PCR reactions were carried out using a CFD-3120 Mini Option detection system (Bio-Red, Hercules, CA, USA). In order to allow the visualization of the PCR products in real time, an SYBR Green RT-PCR kit (SYBR Green I SuperMix, Bio-Red Laboratories Inc., Hercules, CA, USA) was used. The expression of each gene was evaluated in triplicate. The primers used were: E-cadherin (forward: 5′ AAGGGCTTGGATTTTGAGG 3′; reverse: 5′ AGATGGGGGCTTCATTCAC 3′), ICAM-1 (forward: 5′ GCCTGGGGTTGGAGACTAAC 3′; reverse: 5′ CTGTCTTCCCCAATGTCGCT 3′), calretinin (forward: 5′ TATCCAGCAGCTCACCACCTAC 3′; reverse: 5′ GAGAGGTCTGGGAAGGAGTTTC 3′), VEGF (forward: 5′ TGAGACCCTGGTGGACATCT 3′; reverse: 5′ CTCCTATGTGCTGGCTTTGG 3′) and GAPDH (forward: 5′ CACCATCTTCCAGGAGCGAG 3′; reverse: 5′ GGCGGAGATGATGACCCTTT 3′).

### 4.9. Live/Dead Staining

The viability of mesothelial cells in the membrane scaffold was determined using the live/dead viability/cytotoxicity kit. The cell-seeded membranes were washed with PBS and stained with 1 mL staining solution at 37 °C for 15 min. The staining solution was prepared by diluting 3 μL calcein AM and 5 μL ethidium homodimer-1 with 10 mL PBS. The live and dead cells were imaged under a confocal laser scanning microscope (Leica TCS SP8, Wetzlar, Germany) at an excitation/emission wavelength of 494/517 nm and 528/617 nm for live (green) and dead cells (red), respectively.

### 4.10. Cytoskeleton Staining

In order to assess the cytoskeletal structure of the mesothelial cells in the membrane, the cell-seeded membrane was fixed in 10% (*w*/*v*) formaldehyde for 1 h at room temperature. The sample was washed with PBS, and the cell membrane was permeabilized with 0.1% Triton X-100 for 5 min. The samples were stained with 50 μg/mL phalloidin-TRITC solution for 30 min in the dark, and was washed three times with PBS. The cell nuclei were counterstained with 10 μg/mL Hoechst 33258 for 30 min. The cytoskeletal arrangement was observed with a Leica TCS SP8 confocal laser scanning microscope, with the F-actin cytoskeleton showing red fluorescence at an excitation wavelength of 540 nm and an emission wavelength of 570 nm. The nucleus showed blue fluorescence at an excitation wavelength of 340 nm and an emission wavelength of 488 nm.

### 4.11. Immunofluorescence (IF) Staining

For the immunofluorescence (IF) staining of calretinin and E-cadherin, a cell-seeded membrane was cultured for 7 days and fixed in 10% (*w*/*v*) formaldehyde at 4 °C for 1 h. After washing three times in PBST (PBS containing 0.1% Tween 20), the sample was treated with the HyBlock blocking buffer for 1 min in order to block non-specific binding and washed with PBST. The polyclonal primary antibody for calretinin (rabbit) or E-cadherin (rabbit) was added and incubated at 4 °C overnight. After washing with PBST three times for 10 min each, the samples were incubated in fluorescein (FITC)-conjugated AffiniPure goat anti-rabbit IgG (H + L) secondary antibody for 1 h at 37 °C. For nuclear staining, the samples were washed three times in PBST for 10 min each, and were incubated with 100 μg/mL Hoechst 33258 for 30 min at room temperature. After washing with PBST three times, the fluorescein-stained samples were observed by a confocal laser scanning microscope (Leica TCS SP8) at excitation/emission wavelengths of 490 nm/525 nm for fluorescein and 350 nm/461 nm for Hoechst 33258. A semi-quantitative evaluation of calretinin and E-cadherin synthesis by mesothelial cells in the membrane was carried out using ImageJ software.

### 4.12. In Vivo Studies

All of the animal study protocols were approved by the Institutional Animal Care and Use Committee of Chang Gung University (IACUC approval no. CGU106-170; approved on 29 November 2019). Sexually mature, male SD rats weighing between 300 and 380 g were used for the surgery. The SD rats were randomly divided into acellular and cellular groups, and underwent surgery during general anesthesia induced by the inhalation of isoflurane. After depilating the lower abdomen, the abdomen was cleaned with alcohol and betadine solution, followed by making a C-shaped incision of a 5-cm diameter with sterile techniques for laparotomy. The parietal peritoneum was abraded with a sterilized tooth brush for 100 strokes to create a punctate bleeding surface over a 15-mm diameter wound area. The wound in the acellular group was covered with a 2-cm diameter PCL/CS nanofiber membrane sample. The wound in the cellular group was covered with a 2-cm diameter PCL/CS nanofiber + cells sample, which was prepared by seeding 3 × 10^5^ mesothelial cells to a PCL/CS membrane; this was cultured for seven days in vitro. After treatment, the abdominal wall was closed with a 3-0 nylon running suture without creating any trauma on the abdominal wall, and the skin incision was closed with a 3-0 nylon interrupted suture. The animals were sacrificed by CO_2_ inhalation 7 days after surgery, and the abdominal cavities were opened in a U-shaped incision and examined grossly. For the histological examination, the peritoneum tissue specimens from each group were collected and fixed in 10% formaldehyde for 48 h, and then embedded into paraffin. The paraffin sections were prepared in a 5-μm thickness, and were subject to hematoxylin and eosin (H&E) staining for histological evaluation. For the immunohistochemical (IHC) staining, the harvested specimens were fixed in 10% formaldehyde, dehydrated, and embedded in paraffin. Serial sections were deparaffinized and rehydrated. The sections were then washed three times in PBST for 5 min. The non-specific binding sites were blocked with hydrogen peroxide for 10 min, and were washed three times in PBST for 5 min each. The sections were incubated for 60 min at room temperature in a rabbit anti-calretinin polyclonal primary antibody solution or a rabbit anti-E-cadherin polyclonal primary antibody solution in a humid environment. The slides were washed in PBST for 5 min, and HRP Polymer Quanto was applied for 10 min and washed for 5 min. The slide was incubated with a 3-diaminobenzidine (DAB) solution for 1 min for color development, counterstained with hematoxylin for 30 s, and observed under an inverted optical microscope.

### 4.13. Statistical Analysis

All of the data were expressed as the mean ± standard deviation (SD). For the statistical analysis, one-way analysis of variance (ANOVA) was used with the least significant difference (LSD) test to determine significant difference at *p* < 0.05.

## 5. Conclusions

Our study demonstrates the feasibly of the use of electrospun nanofiber membranes as a scaffold for mesothelial cell culture and transplantation. The composite PCL/CS membrane fabricated by the blend electrospinning of CS and PCL can drastically increase membrane hydrophilicity to facilitate cell attachment but can decrease the mechanical strength of the scaffold. Although no change in fiber diameter or porosity was noted, the incorporation of CS substantially influences the cellular response of seeded mesothelial cells. As seen in the in vitro cell culture, rat mesothelial cells proliferate well in both membranes with comparable proliferation rates, but the cells showed a higher attachment rate in PCL/CS with a better maintenance of cell morphology. The attached mesothelial cells also show better phenotypic expression in PCL/CS from qRT-PCR and IF staining, with the upregulated expression of mesothelial specific genes and the higher synthesis rate of the key mesothelial marker proteins E-cadherin and calretinin. In animal studies, the PCL/CS membrane was shown to be feasible as a good vehicle for delivering mesothelial cells, where only scaffold/cell construct but not the scaffold alone could repair damaged mesothelium in rats. After implantation, the cell-seeded PCL/CS scaffold can provide the remodeling of mesothelial cells into a parietal mesothelium by forming a neo-mesothelium tissue similar to native mesothelium from H&E and IHC staining.

## Figures and Tables

**Table 1:** The physical properties of PCL and PCL/CS (mean ± SD, n = 3).

Properties	PCL	PCL/CS
Fiber diameter (nm)	294 ± 110	280 ± 89
Water contact angle (degree)	113.6 ± 2.5	60.9 ± 4.0 *
Porosity (%)	68.3 ± 2.8	74.2 ± 1.3 *
Density (g/cm^3^)	0.484 ± 0.046	0.478 ± 0.188

* *p* < 0.05 compared with PCL.

**Table 2:** The mechanical properties of PCL and PCL/CS nanofiber membranes from tensile testing (mean ± SD, n = 3).

Properties	PCL	PCL/CS
Young’s modulus (MPa)	7.87 ± 0.77	2.34 ± 0.31 *
Ultimate stress (MPa)	1.61 ± 0.10	0.23 ± 0.08 *
Ultimate strain (mm/mm)	1.54 ± 0.32	0.11 ± 0.03 *

* *p* < 0.05 compared with PCL.

## Data Availability

The data presented in this study are available on request from the corresponding author.
